# Impact of Amelogenesis Imperfecta on Junctional Epithelium Structure and Function

**DOI:** 10.3390/biology14070853

**Published:** 2025-07-14

**Authors:** Kevin Lin, Jake Ngu, Susu Uyen Le, Yan Zhang

**Affiliations:** Department of Orofacial Sciences, School of Dentistry, University of California, San Francisco, CA 94143, USA; keviinnn.lin1@gmail.com (K.L.); jake.ngu@ucsf.edu (J.N.); susu.le@ucsf.edu (S.U.L.)

**Keywords:** ameloblasts, enamel matrix formation, amelogenesis imperfecta, the junctional epithelium, internal basal lamina, epithelium barrier, epithelial repair

## Abstract

The junctional epithelium, which lines the inner gingival surface, seals the gingival sulcus to block the infiltration of food debris and pathogens. The junctional epithelium is derived from the reduced enamel epithelium, consisting of late developmental stage ameloblasts and accessory cells. No prior studies have investigated whether defective ameloblast differentiation or enamel matrix formation affects junctional epithelium anatomy or function. Here, we examined the junctional epithelium in mice exhibiting amelogenesis imperfecta due to loss-of-function mutations in the major enamel matrix protein amelogenin (*Amelx*^−/−^) or the critical enamel matrix protease KLK4 (*Klk4*^−/−^). Histological analyses demonstrated altered morphology and cell layer thickness of the junctional epithelium in *Amelx*^−/−^ and *Klk4*^−/−^ mice as compared to *wt*. Immunohistochemistry revealed reduced ODAM, laminin 5, and integrin α6, all of which are critical for the adhesion of the junctional epithelium to the enamel in *Amelx*^−/−^ and *Klk4*^−/−^ mice. Furthermore, we observed altered cell–cell adhesion and increased permeability of Dextran-GFP through the mutants’ junctional epithelium, indicating defective barrier function. Reduced β-catenin and Ki67 at the base of the junctional epithelium in mutants suggest impaired mitotic activity and reduced capacity to replenish continuously desquamated epithelium. These findings highlight the essential role of normal amelogenesis in maintaining junctional epithelium homeostasis.

## Simple Summary

The gingival sulcus, a natural pocket around teeth, is prone to trapping food particles and bacteria, often leading to infection and inflammation that damage the epithelial barrier and the underlying periodontal ligament, cementum, and alveolar bone. If left untreated, this infection can progress to periodontitis, a chronic disease affecting 20–50% of the global population and costing the U.S. USD 154.06 billion in treatment in 2018. The junctional epithelium lines the gingival sulcus, serving as a protective barrier and anchoring the gingiva to the enamel to seal and prevent infiltration by food and pathogens. The junctional epithelium originates from the reduced enamel epithelium, comprising the late developmental stage of ameloblasts, cells that form the enamel tissue. This study investigates whether defective ameloblast differentiation and enamel matrix formation impair junctional epithelium structure and weaken its protective function. In mouse models lacking the key enamel matrix protein amelogenin (*Amelx*^−/−^) or enamel matrix proteinase kallikrein-related peptidase 4 (KLK4) (*Klk4*^−/−^), we observed altered junctional epithelial cell morphology, reduced matrix–cell and cell–cell adhesion, and increased Dextran-GFP penetration, indicating a more permeable barrier and compromised seal. Reduced β-catenin and Ki67 at the junctional epithelium’s base in mutants suggest impaired epithelial regenerative capacity. These findings imply the critical roles of ameloblasts and enamel matrix in the development of healthy gums.

## 1. Introduction

The gingival sulcus is prone to accumulating food debris and bacteria. To protect underlying periodontal tissues from bacterial infiltration, it employs a range of defense mechanisms to restrict pathogen access [1]. A major player in the defensive mechanism of the gingival sulcus is the junctional epithelium (JE). This epithelial layer lines the inner apical surface of the sulcus, anchoring the gingiva to the enamel and sealing the sulcus opening to block microorganisms from penetrating deeper into the periodontal tissues [2,3]. The junctional epithelium serves as a protective barrier for the underlying gingival tissues while permitting the controlled movement of fluids and immune cells [2,4,5].

To seal the pathway between the aggressive oral environment and the periodontal tissues, the tightly interconnected junctional epithelium adheres to the enamel surface of the tooth via an intermediate basement membrane known as the internal basal lamina [6]. The internal basal lamina consists of a dense network of proteins, including laminin 332 [3], odontogenic ameloblast-associated protein (ODAM) [7], amelotin (AMTN) [7], and other glycoproteins.

Laminin 332, also known as laminin 5, is a heterotrimeric glycoprotein, made up of three polypeptide subunits (alpha-3, beta-3, and gamma-2) [7,8]. As a variant specific to epithelial basement membranes, laminin 5 is crucial for adhesion, migration, and proliferation of epithelial cells [7]. It serves as a scaffold for cells and helps maintain the integrity and function of epithelial tissue [9]. The heterotrimeric structure of lamina is specialized for mechanical strength, which is likely important for tightly sealing the periodontal tissues from the oral environment [6]. Additionally, ODAM and AMTN are highly present in the maturation stage of amelogenesis, the junctional epithelium, and the associated internal basal lamina [10]. ODAM and AMTN are part of the secretory calcium-binding phosphoprotein gene cluster. Many genes in this cluster encode proteins responsible for stabilizing calcium and phosphate ions in bodily fluids and facilitating their deposition into the extracellular matrix [9,10]. In the internal basal lamina, ODAM is localized on the surface of junctional epithelial cells, suggesting that they may form molecular assemblies to adhere junctional epithelial cells to the tooth enamel [6]. ODAM also interacts with AMTN in the internal basal lamina between the junctional epithelium and the enamel surface [9].

The internal basal lamina mediates a strong attachment between the junctional epithelium and the tooth enamel surface through hemidesmosomes [11]. In addition, cell–cell interactions are also crucial for the protective function of the junctional epithelium. The junctional epithelial cells are connected through desmosomes, gap junctions, and tight junctions that simultaneously allow fluid and leukocyte movement while acting as a barrier against the ingress of microbes [2,12,13]. Desmosomes are found both in the coronal and apical regions of the junctional epithelium, though in smaller quantities compared to other oral epithelia. Gap junctions are found throughout the junctional epithelium. Larger gap junctions are in the coronal portion, whereas fewer and smaller gap junctions are found at the base of the junctional epithelium. These gap junctions are primarily formed by the key gap-junction-associated protein, connexin 43 [8]. The relatively low number of intercellular junctions allows for wide intercellular spaces to exist between junctional epithelial cells. These spaces serve as reservoirs for polymorphonuclear leukocytes (PMNs) and gingival fluid. PMNs migrate through these spaces and occupy about 1–2% of the space under noninflammatory conditions and about 30% or higher under inflammatory conditions. Gingival fluid also travels through these intercellular spaces, serving as a diffusion pathway for various antibodies, enzymes, and other molecules involved in inflammation to move between the sulcus and periodontal tissues [3].

Upon completion of enamel formation, the inner layer of ameloblasts and papillary layer cells merge to form the reduced enamel epithelium, which covers the enamel surface. During tooth eruption, this reduced enamel epithelium fuses with the oral gingival epithelium, transforming into the junctional epithelium [2,12]. In amelogenesis imperfecta, the morphogenesis of ameloblasts and the production or processing of enamel matrix proteins are significantly impaired [14,15,16,17,18,19,20,21,22,23,24,25,26]. The impact of these defects on the formation of junctional epithelium remains unexplored. Our laboratory maintains two well-characterized mouse models of amelogenesis imperfecta: *Amelx*^−/−^, which lacks amelogenin, and *Klk4*^−/−^, which is deficient in the proteinase kallikrein-related peptidase 4 (KLK4). Amelogenins are the primary enamel matrix proteins produced by secretory ameloblasts, playing a key role in scaffolding during enamel matrix formation [27,28,29]. Kallikrein-related peptidase 4, the predominant proteinase synthesized by maturation ameloblasts, degrades enamel matrix proteins and facilitates matrix calcification [30,31,32]. Thus, we aim to address this knowledge gap using these models.

We hypothesize that loss-of-function mutations in amelogenin or kallikrein-related peptidase 4, which lead to defective ameloblasts, will furthermore differentiate into morphologically and functionally compromised junctional epithelial cells. Our histological, immunohistochemical, and barrier function analyses of junctional epithelial cells in *Amelx*^−/−^ and *Klk4*^−/−^ mice reveal altered morphology, impaired production of internal basal lamina proteins, and compromised barrier function. Elucidating the role of ameloblasts and the enamel matrix in regulating junctional epithelial cell function could enable early identification of individuals at risk for gingival defense deficiencies and inform targeted preventive dental strategies for patients with amelogenesis imperfecta.

## 2. Materials and Methods

### 2.1. Animals

Wild-type (*wt*) C57BL/6J mice were obtained from the Jackson Laboratory. Previously established *Amelx*^−/−^ [14] and *Klk4*^−/−^ [32] mouse lines had loss-of-function mutations in amelogenin and kallikrein-related peptidase 4, respectively. We backcrossed *Amelx*^−/−^ founder with C57BL/6J for four generations to reduce genetic variability since the original *Amelx*^−/−^ was established on C56Bl/6X129/Sv background [14]. All mice were housed in the UCSF animal care facility, a barrier facility accredited by the Association for Assessment and Accreditation of Laboratory Animal Care (AAALAC). No differences in enamel or ameloblast phenotypes were observed between male and female mice in these models; thus, both sexes were included in this study. All experimental procedures were approved by the Institutional Animal Care and Use Committee (IACUC) under protocol AN194670-01.

### 2.2. Histology and Immunohistochemical Staining Assessment

Mice at postnatal week 7 (P7W) were collected following standard IACUC protocols. Briefly, mice were euthanized by CO_2_ inhalation followed by cervical dislocation, then perfusion-fixed with 4% paraformaldehyde (PFA). Hemimandibles were dissected, post-fixed in 4% PFA for 24 h at 4 °C, and subsequently decalcified in 8% EDTA at 4 °C for two weeks, with the EDTA solution changed every other day. The hemimandibles were then processed, embedded in wax, and then sectioned along the sagittal plane or across the first molars. Sections were stained with Hematoxylin and Eosin (H&E) for morphological assessment.

For immunohistochemical staining, the sagittal or transverse sections were boiled in 10 mM citrate buffer (pH 6.0) for 20 min to retrieve antigens. Sections were then incubated with GeneTex Universal Protein Blocking reagent (Genetex Inc., Irvine, CA, USA) for 1 h, followed by overnight incubation with primary antibody at 4 °C. The primary antibody information is provided in Supplement S1. Subsequently, sections were incubated with Alex 594 fluorescein-conjugated or biotin-labeled species-specific IgG for 1 h at room temperature. Sections incubated with Alexa Fluor 594 fluorescein-conjugated IgG were counterstained with 1 µg/mL Hoechst (Life Technologies, South San Francisco, CA, USA) for 5 min. These slides were imaged using a high-speed Leica TCS SP5 spectral confocal microscope (Leica, Wetzlar, Germany). For biotinylated IgG detection, sections were incubated with alkaline phosphatase-conjugated streptavidin (Vector Laboratories, Newark, NJ, USA), visualized using the Alkaline Phosphatase Red Substrate Kit (Vector Laboratories, Newark, USA), counterstained with methyl green, and imaged with a Nikon Eclipse E800 microscope (Nikon, Tokyo, Japan) equipped with an Olympus DP74-CU camera (Olympus, Tokyo, Japan).

### 2.3. Assessment of the Junctional Epithelium Permeability

Six-month-old male or female *wt*, *Amelx*^−/−^, or *Klk4*^−/−^ mice were anesthetized with isoflurane until the toe pinch reflex was absent. Mice were then randomly assigned to either the treatment or control group. In the treatment group, the mouse oral cavity was exposed, and 10 µL of FITC-Dextran-GFP MW 4000, (Sigma, St. Louis, MO, USA) was injected into the gingival sulcus of the right buccal first molar using Hamilton Gastight Neuros syringes. The operator employed a dental loupe to ensure precision throughout the procedure. Control mice received 10 µL of phosphate-buffered saline (PBS) accordingly. Thirty minutes post-injection, mice were euthanized via CO_2_ inhalation followed by cervical dislocation. Hemimandibles were dissected, fixed in 4% paraformaldehyde (PFA) for 24 h at 4 °C, and decalcified in 8% EDTA at 4 °C for two weeks, with the EDTA solution changed every other day. Tissues were processed, embedded in paraffin wax, and sectioned across the first molars. The fluorescence of GFP is typically quenched but structurally preserved following paraffin embedding and processing [33]. To detect the tissue distribution of Dextran-GFP, the tissue sections were incubated with chicken anti-GFP antibody (Aves Labs, Davis, CA, USA) for 1 h, followed by rigorous washing. Sections were then incubated with Alexa Fluor 555-labeled goat anti-chicken IgG (Invitrogen, Waltham, MA, USA), counterstained with 1 µg/mL Hoechst (Invitrogen) for 5 min, and imaged using a Nikon Eclipse E800 microscope. Six animals were analyzed in each group.

## 3. Results

**Figure 1:**
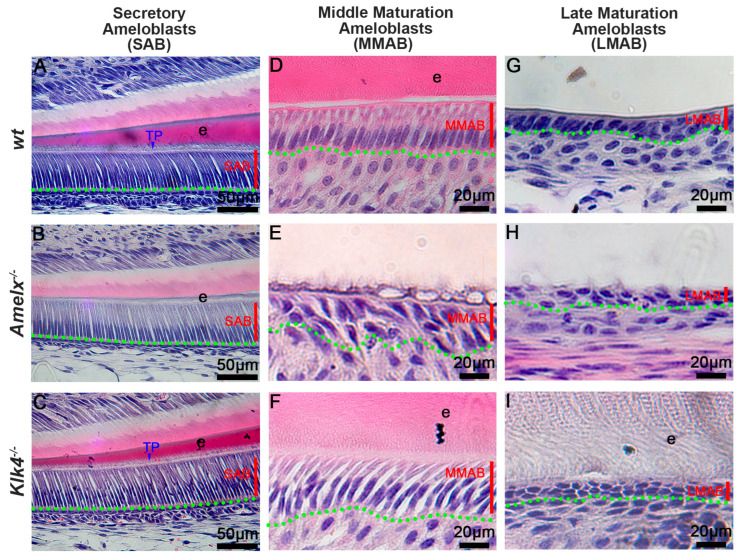
Deficiency in ameloblasts and enamel matrix in *Amelx*^−/−^ and *Klk4*^−/−^ mouse incisors revealed through histological analysis. (**A**) In wild-type (*wt*) mice, secretory ameloblasts (SABs) were elongated, well-organized, and developed a picket fence-like Tomes’ process (TP) at their apical surface, which is essential for the formation of proteinaceous enamel matrix (e) and aligned hydroxyapatite crystals. (**B**) In *Amelx*^−/−^ mice, the Tomes’ process (TP) was poorly developed, and a notably thin enamel matrix layer was detected. (**C**) No notable morphological changes were observed in SABs and enamel matrix in *Klk4*^−/−^ mouse incisors. (**D**) In *wt* mice, maturation ameloblasts (MABs) are reduced in height and establish a distinct boundary with the underlying papillary layer. (**E**) In *Amelx*^−/−^ mouse incisors, MABs organized a multilayered structure, accompanied by a notably thin enamel matrix layer. (**F**) In *Klk4*^−/−^ mouse incisors, the morphology of MABs was largely similar to *wt* controls; however, the enamel matrix (e) exhibited elevated protein retention and expanded intercellular spaces between cells. (**G**) The late stage of *wt* MABs was markedly reduced in height, transitioning into low columnar epithelial cells that fused with the underlying papillary layer cells. Proteins were not detectable in the decalcified enamel matrix. (**H**) At later stages, MABs in *Amelx*^−/−^ mouse incisors became depolarized, flattened in morphology, and formed a multilayered structure. (**I**) In the late stage, MABs in *Klk4*^−/−^ mouse incisors were depolarized, and proteins were detectable in the enamel matrix (e).

The morphology of ameloblasts (ABs) was altered in *Amelx*^−/−^ and *Klk4*^−/−^ mice. Amelogenin and KLK4 are essential enamel matrix proteins and causative genes of amelogenesis imperfecta. Amelogenins are primarily synthesized in secretory ameloblasts (SABs), while KLK4 is mainly produced in maturation-stage ameloblasts (MABs) [14,26,28,32,34,35,36]. Our histological analyses showed that *Klk4*^−/−^ secretory ameloblasts (SABs) (see Figure 1C) closely resembled *wt* SABs (see Figure 1A), whereas *Amelx*^−/−^ SABs were shorter with poorly developed Tomes’ processes (see Figure 1B). As ameloblasts advance to the mid-maturation stage (MMAB), *Amelx*^−/−^ cells became progressively shorter, less polarized, and were associated with a disorganized papillary layer (see Figure 1E) compared to polarized *wt* MMABs (see Figure 1D). In contrast, *Klk4*^−/−^ ameloblasts remained elongated but showed widened intercellular spaces (see Figure 1F). By the late maturation stage, *Amelx*^−/−^ and *Klk4*^−/−^ ameloblasts were both depolarized, with an increasingly indistinct boundary between ameloblasts and the papillary layer (see Figure 1H, I). Compared to *wt* mice, *Amelx*^−/−^ mice exhibited markedly thinner enamel (approximately 10% of healthy enamel thickness), whereas *Klk4*^−/−^ mice developed enamel of normal thickness but with aberrant retention of matrix proteins (see Figure 1I). Since junctional epithelial cells originate from reduced ameloblasts and papillary layer cells in the subsequent developmental stage, we next sought to investigate whether the altered ameloblasts and enamel matrix in *Amelx*^−/−^ and *Klk4*^−/−^ mice affect the junctional epithelial cell phenotype and functions.

**Figure 2:**
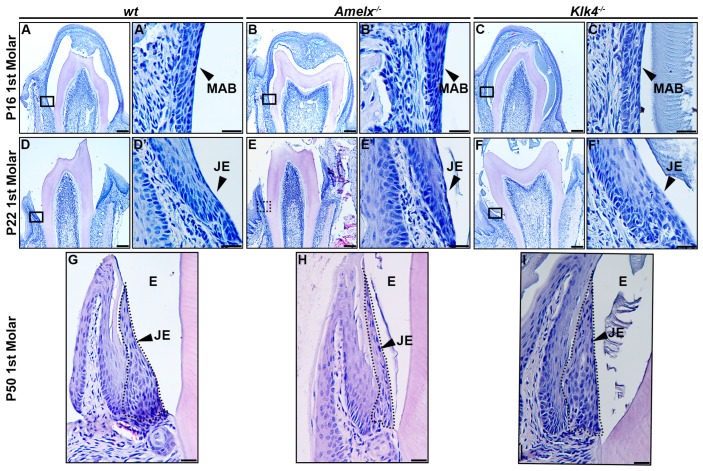
Morphological changes in the junctional epithelium of *Amelx*^−/−^ and *Klk4*^−/−^ mouse molars. (**A**) H&E staining of a representative transverse section of a *wt* mouse first molar at P16 showed that the erupting tooth positioned the reduced ameloblasts and papillary layer toward the oral epithelium. (**A’**) A magnified view of the cells in the boxed region of panel A showed organized and flattened reduced ameloblast epithelial cells. (**B**) H&E staining of a representative transverse section of an *Amelx*^−/−^ mouse first molar at P16 revealed a narrower enamel space between the ameloblast and dentin layers, attributed to a thin enamel layer. (**B’**) A magnified view of the boxed region of panel B showed irregularly shaped, depolarized ameloblasts with enlarged nuclei. (**C**) H&E staining of a representative transverse section of a *Klk4*^−/−^ mouse first molar at P16 showed the retained proteins in the enamel space. (**C’**) A magnified view of the boxed region in panel C showed a less organized ameloblast cell layer. (**D**) H&E staining of a cross-section from the first molar of a *wt* mouse at P22. (**D’**) A magnified view of the boxed region of panel D showed a junctional epithelium with cells exhibiting more elongated shapes and reduced cytosol at the apical portion. (**E**) H&E staining of a transverse section from the first molar of an *Amelx*^−/−^ mouse at P22. (**E’**) A magnified view of the boxed region in panel E showed disorganized junctional epithelial cells. (**F**) H&E staining of a transverse section from the first molar of a *Klk4*^−/−^ mouse at P22. (**F’**) A magnified view of the boxed region in panel F showed less organized junctional epithelial cells with abundant cytosol and increased intercellular spacing. (**G**) H&E staining of a transverse section from the first molar of a *wt* mouse at P50. (**H**) H&E staining of a transverse section from the first molar of an *Amelx*^−/−^ mouse at P50 showed a thin junctional epithelium layer. (**I**) H&E staining of a transverse section from the first molar of a *Klk4*^−/−^ mouse at P50 showed junctional epithelial cells with less elongated shapes at the apical surface compared to controls, as indicated by the black arrow.

The morphology of junctional epithelial cells in *Amelx*^−/−^ and *Klk4*^−/−^ mouse molars differs from that of *wt* junctional epithelial cells. The developmental trajectory of ameloblasts and the enamel matrix has been thoroughly characterized in mouse molars [34]. At postnatal day 16 (P16), the first molars in mice were poised for tooth crown eruption. At this stage, ameloblasts fused with the papillary layer, forming squamous, multilayered epithelial cells that began interacting with the oral mucosal epithelium in the oral cavity (see Figure 2A–C). At higher magnification, ameloblasts in the molars displayed phenotypes consistent with those observed in mouse incisors: late-stage *Amelx*^−/−^ ameloblasts appeared less organized and thinner, while *Klk4*^−/−^ molar ameloblasts showed increased intercellular spacing and interacted with retained enamel matrix proteins (see Figure 2A’–C’).

At postnatal day 22 (P22), the junctional epithelium forms immediately following tooth eruption to anchor the gingiva to the enamel surface. In the gingiva of wild-type mice, junctional epithelial cells at the apical region exhibited elongated, slender nuclei and a cytosol with widened intracellular spaces. In contrast, cells at the basolateral region displayed larger, oval nuclei (see Figure 2D’). These cells become increasingly elongated as they progress toward the apical surface and coronal end (see Figure 2D,D’). In *Amelx*^−/−^ mice, certain junctional epithelial cells exhibited densely stained nuclei and increased cytosolic volume. As they progressed toward the apical surface and coronal end, these cells appeared to have fewer irregular outlines (see Figure 2E,E’). In *Klk4*^−/−^ mice, junctional epithelial cells were larger, exhibited wider intercellular spaces, and formed a thicker epithelial layer (see Figure 2F,F’).

In P50 *wt* mice, the junctional epithelial cells retained the same characteristics observed at P22 (see Figure 2G). In P50 *Amelx*^−/−^ mice, the junctional epithelium layer was thinner, with cells exhibiting greater irregularity and reduced cytosolic content (see Figure 2H). In P50 *Klk4*^−/−^ mice, the junctional epithelial cells displayed increased cytosolic content, with cells toward the apical surface and coronal portion appearing less elongated (see Figure 2I).

**Figure 3:**
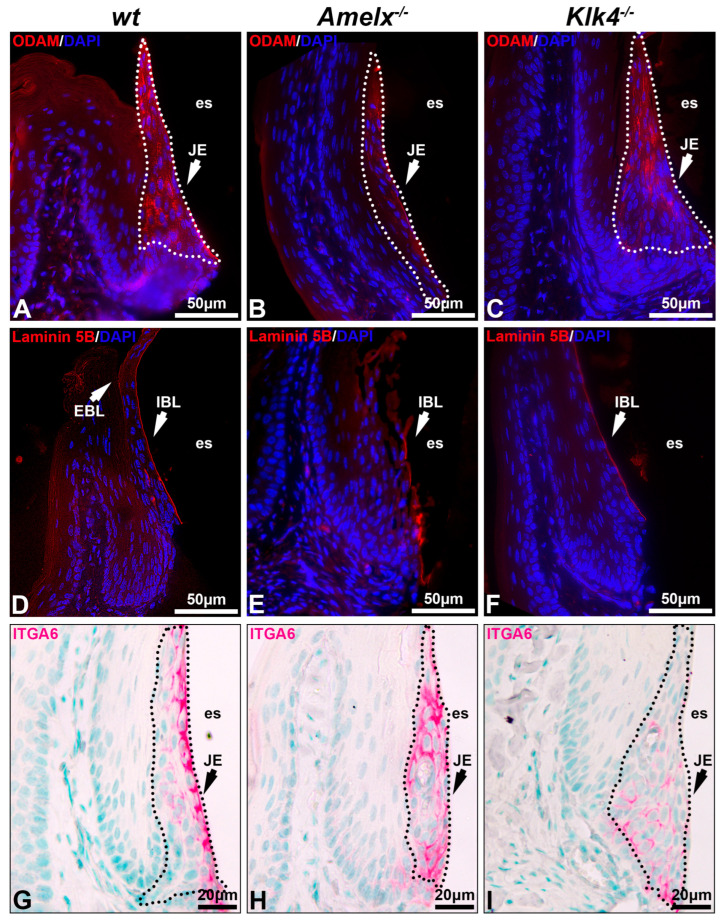
Proteins associated with internal basal lamina and cell–extracellular matrix interaction in *Amelx*^−/−^ and *Klk4*^−/−^ mouse junctional epithelium. Immunostaining of transverse sections from mouse first molars was performed to visualize key components of the internal basal lamina, including ODAM (**A**–**C**), laminin 5B (**D**–**F**), and integrin α6 (**G**–**I**), the cell surface receptor for laminin 5B.

The interaction between junctional epithelial cells and internal basal lamina is impaired in the gingiva of *Amelx*^−/−^ and *Klk4*^−/−^ mice. Junctional epithelial cells secrete molecules that form the dense network of internal basal membrane, mediating gingival attachment to the enamel surface, sealing the gingival pocket, and preventing bacterial invasion [2,3]. We investigated the impacts of amelogenin or KLK4 loss-of-function on junctional epithelial cell functions by analyzing two key basal lamina proteins, ODAM and laminin 5B, as well as integrin alpha 6 (ITGA6), the cell surface receptor for laminin 5B [37]. In the *wt* mouse gingiva, ODAM was detected in junctional epithelial cells and adjacent internal basal lamina (IBL) (see Figure 3A), laminin 5B was predominantly present in the internal basal lamina and visible on the external basal lamina (EBL) (see Figure 3D), and ITGA6 was well organized on the junctional epithelial cell membrane, with a strong concentration at their apical surface (see Figure 3G). Junctional epithelial cells in *Amelx*^−/−^ mice exhibited reduced ODAM level (see Figure 3B). Laminin 5B and ITGA6 were less densely distributed and less concentrated along the apical side of the junctional epithelium (see Figure 3E,H). In *Klk4*^−/−^ mice, ODAM was barely deposited along the apical side of the gingiva (see Figure 3C), whereas laminin 5B was present along the apical surface of the junctional epithelium, albeit in reduced abundance (see Figure 3F). ITGA6-positive immunostaining was observed across all layers of the junctional epithelial cells (see Figure 3I). No EBL was detectable in *Amelx*^−/−^ and *Klk4*^−/−^ mouse gingiva. These findings indicate that proper ameloblast differentiation is crucial for junctional epithelial cells to form the internal basal lamina, which anchors the gingiva to the enamel surface.

**Figure 4:**
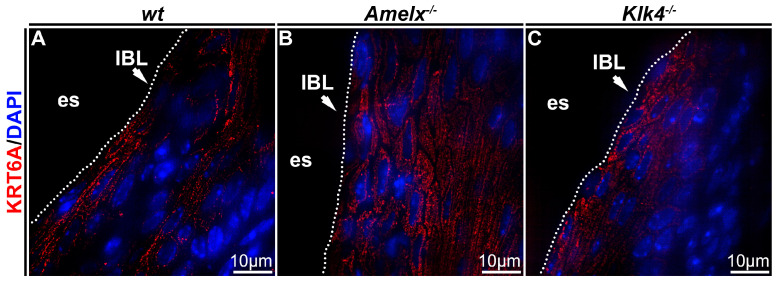
Immunostaining images illustrate the distribution of cytokeratin KRT6A in the junctional epithelium of *wt*, *Amelx*^−/−^ and *Klk4*^−/−^ mouse gingiva. The distribution of KRT6A (in red) in *wt* junctional epithelial cells was organized in the cytosol, aligning with the long axis of cells at the apical region of the junctional epithelium (**A**). The KRT6A meshwork in irregularly shaped *Amelx^−/−^* junctional epithelial cells was markedly disorganized (**B**). The KRT6A distribution pattern in *Klk4^−/−^* junctional epithelial cells was less uniform compared to that in *wt* cells (**C**).

Cytokeratin organization was altered in *Amelx*^−/−^ and *Klk4*^−/−^ mouse junctional epithelial cells. Cytokeratin is crucial for epithelial cells because it provides structural support, maintains cell shape, and facilitates motility and responses to environmental cues [38]. To investigate cytokeratin in the junctional epithelium of those mouse models, we focused on cytokeratin 6A, a cytokeratin previously identified in human junctional epithelial cells [4,39,40]. In *wt* mice, immunostaining demonstrated that the KRT6A network was densely and uniformly distributed between nuclei and plasma membranes, aligning with the elongated, oval-shaped architecture of junctional epithelial cells (see Figure 4A). In irregularly shaped *Amelx*^−/−^ junctional epithelial cells, KRT6A formed a disorganized cytokeratin meshwork marked by unevenly distributed filaments, patchy staining, and a lack of smooth, consistent contours (see Figure 4B). In *Klk4*^−/−^ mice, KRT6A distribution in junctional epithelial cells was sparse, exhibiting patchy staining and irregular patterns.

**Figure 5:**
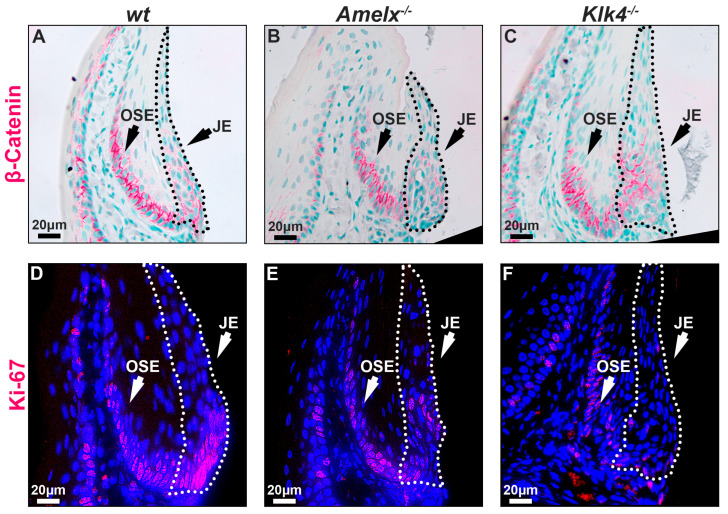
Immunostaining images illustrate the distribution of β-catenin and Ki67 in the gingiva of *wt*, *Amelx*^−/−^, and *Klk4*^−/−^ mice. The junctional epithelium was enclosed by dashed lines. The immunostaining pattern of β-catenin in the OSE region was similar across the three mouse models. However, β-catenin was predominantly localized at the base of the *wt* junctional epithelium (**A**), less abundant in *Amelx^−/−^* cells (**B**), and reduced toward the apical portion of the *Klk4^−/−^* junctional epithelium (**C**). The immunostaining signal of Ki67 was concentrated at the base of the *wt* junctional epithelium (**D**), reduced in *Amelx^−/−^* cells (**E**), and less prominent, particularly in the apical portion of the *Klk4^−/−^* junctional epithelium (**F**).

The replenishing ability of the junctional epithelial cells in *Amelx*^−/−^ and *Klk4*^−/−^ mouse gingiva was reduced. The capacity of epithelial cells to self-renew is vital for maintaining tissue homeostasis, particularly in regions subject to ongoing constant wear and tear [41]. Epithelial tissues, including the junctional epithelium, depend on ongoing renewal to preserve their structural integrity and functionality. Previous research highlights the Wnt/β-catenin signaling pathway as essential for cell proliferation and migration, facilitating the self-renewal of junctional epithelial cells [42,43]. Therefore, we examined the distribution pattern of β-catenin and Ki67 in the junctional epithelium to understand the mitotic dynamics in *Amelx*^−/−^ and *Klk4*^−/−^ mouse gingiva. Ki67 is recognized as a biomarker of cell proliferation [44,45]. Notably, we observed that β-catenin and Ki67 positive immunostaining signals were co-localized at the base of the junctional epithelium and oral sulcular epithelium (OSE). However, the signal intensity at the base of the OSE was consistent across all three mouse models, suggesting that the loss-of-function of amelogenin and KLK4 did not affect the dynamics of OSE. Within the junctional epithelium in *wt* gingiva, β-catenin and Ki67 exhibited a polarized distribution, extending from the base toward the apical and coronal regions (see Figure 5A,D). In the junctional epithelium of *Amelx*^−/−^ and *Klk4*^−/−^ mouse gingiva, β-catenin did not exhibit the polarized distribution pattern that occurred in *wt* controls (see Figure 5B,C). In particular, the localization of β-catenin-positive cells within the junctional epithelial tissue in the gingiva of *Klk4^−/−^* mice is less detectable apically compared to wild-type (*wt*) and *Amelx^−/−^* mice. Fewer Ki67-labeled proliferating cells were observed at the base of the junctional epithelium in *Amelx*^−/−^ and *Klk4*^−/−^ mouse gingiva (see Figure 5C,F).

These findings suggest that the deletion of enamel matrix-specific genes, amelogenin or KLK4, impairs the ability of self-renewal and repair of the damaged junctional epithelial cells at the apical surface.

**Figure 6:**
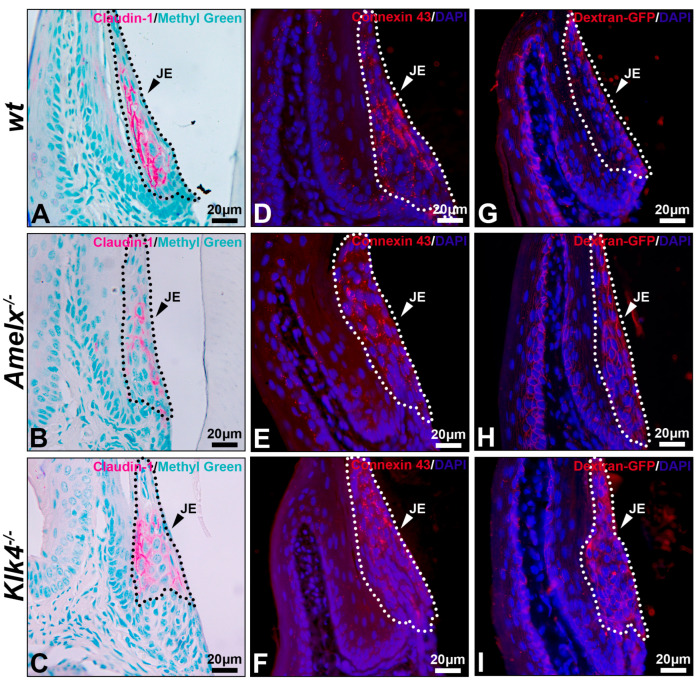
Immunostaining images illustrate the distribution of claudin-1, connexin 43, and dextran-GFP in the junctional epithelium of *wt*, *Amelx*^−/−^ and *Klk4*^−/−^ mouse gingiva. The junctional epithelium (JE) was enclosed by dashed lines. Compared to *wt* (**A**), the immunostaining signal of claudin 1 was reduced in *Amelx^−/−^* junctional epithelium (**B**) and was shifted toward the apical portion in *Klk4^−/−^* junctional epithelium (**C**). The immunostaining signal of connexin 43 was primarily localized in the central portion of the *wt* junctional epithelium (**D**), became more dispersed in *Amelx^−/−^* junctional epithelium (**E**), and shifted toward the coronal portion in *Klk4^−/−^* junctional epithelium (**F**). A limited amount of Dextran-GFP was immunostained beneath the surface of *wt* junctional epithelium (**G**), while an increased amount of Dextran-GFP was detected in the *Amelx^−/−^* and *Klk4^−/−^* junctional epithelium (**H**,**I**).

Permeability of the junctional epithelium was compromised in the gingiva of *Amelx*^−/−^ and *Klk4*^−/−^ mice. Like all epithelial tissues, the junctional epithelium acts as a protective barrier for underlying tissues. Yet, its intercellular spaces are wider than those in other epithelial layers, supporting its unique functions, such as fluid exchange, immune cell migration, and rapid tissue remodeling [46,47]. We investigated the intercellular adhesion molecules claudin-1, a biomarker of the tight junction, and connexin 43, a biomarker of gap junction, in the junctional epithelium. In general, tight junctions were rarely found on the apical surface of junctional epithelium and could only be observed in the middle region of the junctional epithelium [4]. Indeed, in the *wt* mice, claudin-1 is predominantly and uniformly distributed on the lateral surfaces of junctional epithelial cells adjacent to the external basal lamina, rather than on the basal or apical surfaces (see Figure 6A). This distribution pattern aligns with findings previously reported [4].

In both *Amelx*^−/−^ and *Klk4*^−/−^ junctional epithelium, claudin-1 immunostaining was reduced and predominantly distributed toward the coronal portion of this tissue (see Figure 6B,C). Connexin 43 forms gap junctions and hemichannels to facilitate intercellular exchange of small molecules, such as ions, second messengers, and metabolites [48]. Connexin 43 was uniformly distributed throughout the *wt* junctional epithelium. The positive staining appeared as a punctate pattern, consistent with previous descriptions [49] (see Figure 6D), whereas it was predominantly localized toward the coronal region in the *Amelx*^−/−^ and *Klk4*^−/−^ junctional epithelium (see Figure 6E,F).

Thirty minutes after dextran-GFP injection, the GFP-positive signal was predominantly restricted to the apical surface of the wild-type junctional epithelium (see Figure 6G). Conversely, elevated GFP signal was detected throughout the junctional epithelial layers in the *Amelx*^−/−^ and *Klk4*^−/−^ gingiva (see Figure 6H,I). The immunostaining images from the PBS injection group are included in Supplement S2.

## 4. Discussion

Amelogenesis imperfecta is a hereditary disorder that disrupts tooth enamel development, primarily due to mutations in genes critical for enamel formation, such as amelogenin and kallikrein-related peptidase 4. These mutations result in abnormal ameloblast morphology and defective enamel tissue, particularly in the final stages of development, as illustrated in Figure 1. The link between amelogenesis imperfecta and increased susceptibility to dental caries has been well documented [50,51,52,53]. During tooth eruption, oral epithelial cells fuse with reduced enamel epithelial cells, the late-stage remnants of enamel-forming ameloblasts. These reduced enamel epithelial cells subsequently differentiate into primary junctional epithelial cells, a crucial structure for maintaining periodontal health [2,4,40]. However, little research has explored whether amelogenesis imperfecta impacts the morphology and function of junctional epithelial cells. Using mouse models with defective ameloblast differentiation and enamel formation, we showed that such amelogenesis impairments disrupt the morphology, barrier function, and regenerative capacity of junctional epithelial cells, hindering their ability to replace exfoliated or damaged gingival epithelium.

In mouse models of amelogenesis imperfecta, the morphological changes of junctional epithelial cells were noticeable, including less elongated cell shapes, increased cytosolic volume, and variation of the number of cell layers. As a unique tissue, the junctional epithelium balances epithelial barrier function with flexible permeability [4]. These changes in *Amelx*^−/−^ and *Klk4*^−/−^ junctional epithelial cells may disrupt the delicate equilibrium between barrier function and permeability, thus compromising the protective role of the junctional epithelium in these mice. For instance, the enlarged cell body and increased thickness of the junctional epithelium in *Klk4*^−/−^ mice may influence the immune cell response to bacteria persisting in the gingival sulcus.

**Figure 7:**
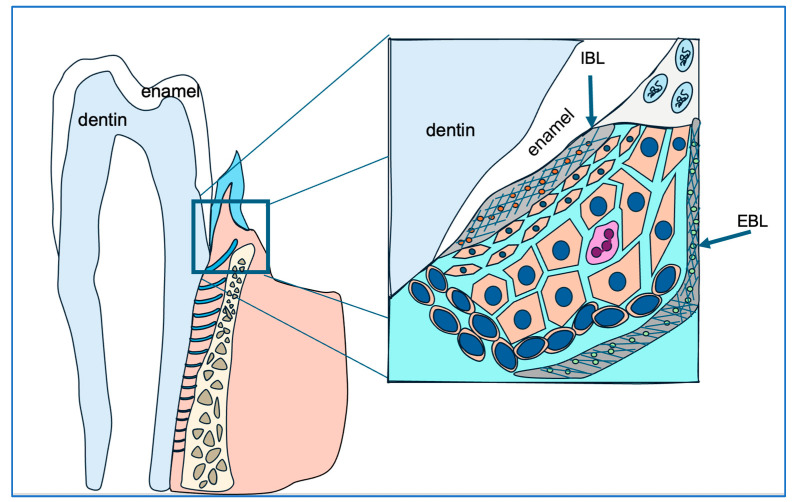
This diagram illustrates the strategic positioning of junctional epithelial cells (cells in orange) in safeguarding gingival and periodontal tissue health. The junctional epithelial cells secrete matrix proteins to construct the internal basal lamina (IBL), which anchors the junctional epithelium to the enamel surface and seals the gingival sulcus, preventing bacterial infiltration.

Junctional epithelial cells construct the internal basal lamina, a special basement membrane, by synthesizing and secreting a unique blend of extracellular matrix components, including ODAM, laminin 5, amelotin, and SCPPPQ1, among others [54,55,56]. This anatomical structure anchors the gingiva to the enamel surface, blocking oral cavity contents and pathogens from infiltrating periodontal supporting tissues (see Figure 7). Using available antibodies, we examined the distribution of ODAM, laminin 5B, and integrin 6A, the cell surface receptor for laminin 5B in *Amelx*^−/−^ and *Klk4*^−/−^ mouse gingiva. Both ODAM and laminin 5B are secretory proteins that contribute to the formation of the internal basal lamina. Our findings suggest that ameloblast abnormalities disrupt the ability of junctional epithelial cells to synthesize and/or secrete proteins essential for matrix–cell adhesion.

Additionally, hypoplastic, hypomineralized, and hypomature enamel matrix may further impair the matrix–matrix–cell adhesion network, further weakening the gingival sulcus seal and potentially increasing susceptibility to bacterial infiltration.

Previous research has shown that ODAM mediates the attachment of the junctional epithelium to the enamel surface by interacting with Rho guanine nucleotide exchange factor 5 (ARHGEF5) to activate RhoA signaling in the junctional epithelium and subsequently organize the cytoskeleton [55]. In *wt* junctional epithelial cells, the highly organized internal basal lamina and KRT6A signify robust matrix–cell interactions that sustain cellular homeostasis. Interactions between extracellular matrix proteins and integrins may transmit adhesion signals to KRT6A, thereby supporting the development of cytokeratins essential for strong mechanical stability, efficient protein transport, and dynamic cell migration and integration within the junctional epithelium.

The junctional epithelium utilizes a rapid turnover rate as one of the protective mechanisms, promoting the exfoliation of epithelial cells to maintain a dynamic and effective barrier. Studies indicate that the apical side of junctional epithelial cells regenerates every 4–6 days [52]. Our immunostaining detected β-catenin, a Wnt signaling component, and Ki67, a proliferation marker, at the junctional epithelium’s base. These are recognized niche-harboring slow-cycling stem cells and highly mitotic cells that supply cells for apical and coronal migration to replace desquamated cells during turnover [42]. Our analyses reveal that amelogenesis imperfecta reduces the pool of mitotic cells available for tissue regeneration, potentially undermining the epithelium’s protective functions and hindering its capacity to maintain a sealed gingival sulcus.

Yamamoto’s research, using a bioengineered tooth system, demonstrates that the junctional epithelium which forms after eruption originates from the dental epithelium [46]. However, further research indicates that junctional epithelial cells are progressively replaced by gingival sulcular epithelial cells, with near-complete substitution occurring within approximately 200 days [26,57,58,59]. This rapid turnover highlights the dynamic nature of junctional epithelium and may lead to a shift in cellular origin, potentially disconnecting secondary junctional epithelial cells from the reduced enamel epithelium. Examining whether junctional epithelial cells in *Amelx*^−/−^ and *Klk4*^−/−^ mice aged over 200 days exhibit the same phenotype as primary junctional epithelial cells would be highly informative. Such a study could verify if the gingival junctional epithelium in older mice, predominantly composed of sulcular-derived cells, retains normal morphology and function, indicating a robust restorative mechanism independent of its original dental epithelial source. These insights could significantly advance our understanding of epithelial regeneration and inform the development of treatment strategies for individuals with amelogenesis imperfecta. This study provides evidence to support the development of preventive strategies, such as strengthening gingival attachment to the tooth surface in *Amelx*^−/−^ AI patients and promoting OSE stem cell-derived epithelial cells to replace primary junctional epithelial cells in *Amelx*^−/−^ and *Klk4*^−/−^ AI patients.

## 5. Conclusions

Using mouse models of amelogenesis imperfecta with defective ameloblast differentiation and enamel formation, we show that these defects impair the morphology, barrier function, and regenerative capacity of junctional epithelial cells. This study highlights the risk of developing periodontal disease in individuals with amelogenesis imperfecta and outlines preventive measures for early intervention. Patients with amelogenesis imperfecta typically first visit a pediatric dentist due to enamel deficiencies. The findings underscore the importance of early prevention and management strategies for periodontal disease.

## Data Availability

The data supporting the findings of this study are available within the article and its Supplementary Materials. Additional data are available from the corresponding author upon request.

## Supplementary Materials

The following supporting information can be downloaded at: https://www.mdpi.com/article/10.3390/biology14070853/s1.
