# Supplementary material for: Impact of Amelogenesis Imperfecta on Junctional Epithelium Structure and Function
Source: Biology (Basel). 2025 Jul 14;14(7):853. doi: 10.3390/biology14070853 (PMC12292820; doi:10.3390/biology14070853)
Supplement: Supplementary file 1 [file biology-14-00853-s001.zip › biology-3661296-supplementary.pdf]

Supplement S1: Primary antibodies: Rabbit-anti-Claudin 1 antibody, abcam, Cat: ab15098, dilution factor: 1:450; Rabbit-anti-Connexin 43 antibody, Novus Biologicals, Cat: NBP2-38234, dilution factor: 1:150; Rabbit-anti-Ki67 antibody, abcam, Cat: ab15580, dilution factor: 1:300; Rabbit-anti-β-catenin antibody, abcam, Cat: ab51032, dilution factor: 1:150; Rabbit-anti-KRT6A antibody, Lifespan Biosciences, Cat: LS-B13811-50, dilution factor: 1:500; Rabbit-anti-KRT14 antibody, Proteintach, Cat: 82824-1-RR, dilution factor 1:500; Rabbit-anti-ODAM antibody, Proteintech, Cat: 16509-1, dilution factor: 1:400; Rabbit-anti-Laminin 5 antibody, abcam, Cat: ab14509, dilution factor: 1:250; Rabbit-anti-integrin α6 antibody, Proteintech, Cat: 27189-1, dilution factor 1:600.

Supplement S2:

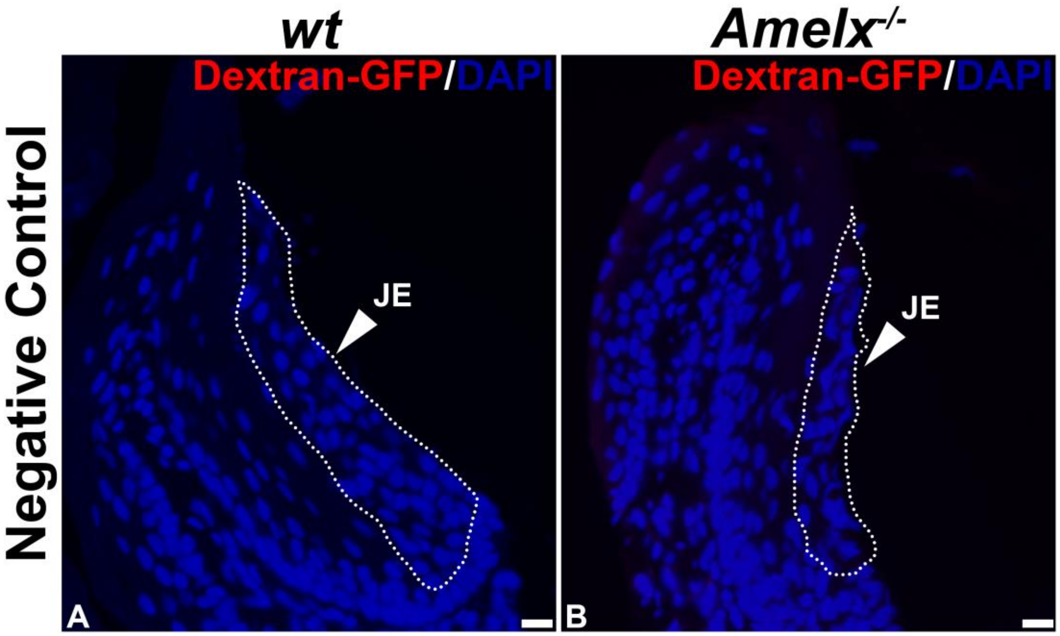

Supplement S2: In the gingiva injected with PBS, anti-GFP antibody immunostaining showed no positive (in red) signal. Scale bars: 20 μm.
